# Improving Outcomes and Preventing Complications in Cranial Base Surgery

**DOI:** 10.3390/brainsci14111045

**Published:** 2024-10-23

**Authors:** Nicola Montano, Renata Martinelli, Joao Paulo Almeida

**Affiliations:** 1 Department of Neuroscience, Neurosurgery Section, Università Cattolica del Sacro Cuore, 00168 Rome, Italy; renata.martinelli01@icatt.it; 2 Department of Neurological Surgery, Mayo Clinic, Jacksonville, FL 32224, USA; almeida.joao@mayo.edu

## 1. Introduction

Skull base surgery has evolved remarkably since the pioneering techniques of early 20th-century surgeons, such as Schloffer and Cushing, who laid the foundation for transcranial and transnasal approaches. Over the decades, advancements in anatomical understanding and surgical technology have transformed these rudimentary methods into sophisticated, minimally invasive procedures. The introduction of endoscopic techniques, intraoperative neuromonitoring, and advanced imaging has dramatically improved patient outcomes, reducing morbidity and mortality in complex cranial base surgeries.

A multidisciplinary approach, combining neurology, radiology, oncology, and electrophysiology, is now essential for addressing the anatomical and functional challenges inherent to this field. As these innovations continue to progress, skull base surgery is poised to deliver even more refined patient-centered care, with a focus on improved outcomes and long-term quality of life.

As the field continues to evolve, the importance of scientific research and publications in driving innovation cannot be overstated, and continuous research efforts ensure that surgeons stay at the cutting edge of these complex procedures, fostering global collaboration and contributing to overall improvement in patient care and quality of life. In this context, the initiative to publish this Special Issue on the topic of improving outcomes and preventing complications in cranial base surgery is a key example of how important it is to stimulate the sharing of surgical knowledge and techniques in such a complex field.

As illustrated by Toader et al. (contribution 1), who not only revisited the past but also brilliantly highlighted the future potential of skull base surgery, the possible fields of application and research for the developed techniques are wide. Among the most common is the endoscopic endonasal approach, widely employed in the surgical treatment of pituitary adenomas, craniopharyngiomas, and rare intra- and parasellar lesions. Additionally, posterior cranial fossa approaches, such as the retrosigmoid approach, have been utilized in the management of different functional and oncological pathologies, as described in several articles published in this Special Issue. Lastly, there are more rarely used approaches, such as the cranio-orbito-zygomatic approach, and structures like the olfactory groove and jugular foramen, which demand specific surgical techniques that have been thoroughly explored and described in some of the texts included in this Issue.

## 2. Discussion

Several articles have explored pituitary pathology, predominantly treated via the endoscopic endonasal approach (EEA). Porras et al. (contribution 2) analyzed the most common complications and the associated pre- and intraoperative factors. The most frequent complication, CSF leak, has significantly decreased over time: from as high as 40% in early series to as low as 2.9% in recent studies, thanks to advances in endoscopic visualization and dural closure techniques. Preoperative factors such as sex, age, BMI, prior radiation therapy, and previous surgeries were not significant in increasing the risk of CSF leak, while intraoperative factors, particularly the presence of a high-flow fistula, were found to be more critical, and vascularized flap reconstruction is recommended over free-tissue grafts in such cases. The use of lumbar drainage remains highly debated in the literature, although a recent study showed that prophylactic lumbar drainage reduced CSF leak rates from 21.2% to 8.2% [1]. Other EEA-associated complications include damage to the optic and cranial nerves (with abducens nerve palsy being most common), panhypopituitarism, ICA injuries, and meningitis. Notably, the authors report that the literature does not show an association between the choice or duration of antibiotics and postoperative meningitis rates. In conclusion, while advances in EEA techniques have greatly reduced complications like CSF leaks, careful consideration of intraoperative factors remains crucial for optimizing outcomes.

Figueredo et al. (contribution 3) also conducted a systematic review and meta-analysis comparing the EEA and open transcranial approach (TCA) in the treatment of craniopharyngiomas. Despite the significant heterogeneity in the studies analyzed, mainly due to varying classifications of infundibular or hypothalamic involvement in reporting postoperative outcomes, several key findings were demonstrated: EEA was associated with higher rates of gross total resection and lower rates of postoperative hypopituitarism and permanent diabetes insipidus compared to TCA. Additionally, the EEA group showed a higher rate of visual improvement, although CSF leaks occurred more frequently, and recurrence rates were lower in the EEA group. Therefore, as EEA shows clear advantages over the transcranial approach for craniopharyngiomas, the increased risk of CSF leaks remains a key consideration in surgical planning.

In the context of pituitary adenomas, Solari et al. (contribution 4) emphasized the importance of selecting between the endoscopic and transcranial approaches, particularly in challenging cases such as giant adenomas, which are defined as having a maximum diameter exceeding 4 cm. They focused on the importance of not solely considering tumor size in the surgical planning phase, but also, and more importantly, the extent of supra- and parasellar extension. These lesions exhibit specific characteristics, such as vertical extension with a lower risk of neurovascular injury; however, gross tumor removal is more difficult, and intralesional hemorrhages frequently occur. Therefore, tumor size alone is not necessarily indicative of the appropriate surgical approach, as summarized by the authors: ‘size doesn’t matter’.

While pituitary pathology is often associated with cavernous sinus invasion, and surgical techniques and outcomes in this region are extensively discussed in the literature, parasellar dural infiltration and its impact on pituitary surgery outcomes are significantly less studied. Recent studies, however, have suggested a role for endoscopic resection of the medial wall of the cavernous sinus in selected cases. This was the focus of a review conducted by de Macêdo Filho et al. (contribution 5), based on 5 studies analyzing a total of 208 patients, with a prevalence of medial wall invasion of the cavernous sinus (CS) reaching up to 36.7%.

Techniques for resecting the medial wall of the CS via the endoscopic endonasal approach include the ‘anterior to posterior’ opening of the anterior cavernous sinus wall and the ‘medial to lateral’ opening of the inferior intercavernous sinus technique. As reported by the authors, resection of the medial wall of the CS in selected cases of pituitary adenomas has been associated with high rates of disease control and positive short-term outcomes. Despite previous concerns regarding higher complication rates, this procedure has demonstrated low morbidity and has proven especially useful in tumors extending to the medial wall of the CS and in cases of recurrent non-functional adenomas with a close relationship to the cavernous ICA or clear infiltration of the medial wall.

Yan et al. (contribution 6) also explored a specific and often underestimated aspect of Pituitary Neuroendocrine Tumors (Pit-NETs), namely hypogonadism. Their review highlights the relative scarcity of studies on gonadal axis function, making it difficult to determine the effectiveness of testosterone levels as a reliable indicator of clinical hypogonadism (according to current guidelines [2], the diagnosis of hypogonadism in adult men requires both low serum testosterone levels and at least one symptom of androgen deficiency). More importantly, they underscore the lack of studies specifically evaluating the efficacy of medical and surgical therapies for this dysfunction, which affects more than 50% of male patients with pituitary macroadenomas and has a significant impact on the quality of life of these patients [3].

Among the decision-making challenges in skull base surgery is often the treatment, predominantly endoscopic, of cystic lesions of intrasellar origin, such as Rathke’s cleft cysts (RCC). Focusing on these, Millesi et al. (contribution 7) conducted a brilliant comparative study between simple fenestration (SF) and complete cyst wall resection (CWR) in 39 patients, of whom 33 underwent SF and 6 underwent CWR. Notably, the authors reported some statistically significant results, including a significantly lower rate of postoperative hypopituitarism in the SF group. Regarding the recurrence rate at a mean follow-up of 58 months, the type of procedure performed did not show a statistically significant difference. Additionally, in line with the literature, the authors found that closed reconstruction of the sellar floor was not a conclusive factor in developing recurrence [4].

Some of the published articles are particularly useful for understanding the technical aspects of endoscopic skull base surgery (ESBS). In their narrative review, Jarmula et al. (contribution 8) discuss the history and development of ESBS, current visualization techniques, and future innovations. Current state-of-the-art endoscopy uses ultra-HD/4K image resolution and 3D vision, while fluorescence agents have shown success in identifying residual tumor tissue, and contrast-enhanced ultrasonography has proven helpful in detecting vascular structures. The use of neuronavigation with electromagnetic tracking, along with advances in virtual reality and augmented reality, provides surgeons with more patient-specific anatomical information for surgical planning and execution. These innovations require continuous updating by neurosurgeons, and the literature needs to describe them to disseminate and stimulate learning.

Regarding innovation, special attention should be given to the issue highlighted by Rech et al. (contribution 9), who explored the potential role of machine learning (ML) models to predict pituitary surgery outcomes. They conducted a review of 20 studies following 2 guidelines: the PRISMA checklist and TRIPOD Adherence Form [5], aiming for consistency and transparency. By providing rationales and highlighting the importance of some of the most poorly reported items in TRIPOD, this review offers valuable insights for future systematic reviews and the development and validation of ML models. This is the first systematic review to assess report completeness concerning ML in neurosurgery.

The posterior cranial fossa is an anatomically complex region and is likely the most fascinating compartment in neurosurgery due to the technical challenges it presents. These include intricate approaches for the treatment of both oncological pathologies, such as cerebellopontine angle (CPA) tumors, and functional conditions, with trigeminal neuralgia and hemifacial spasm being prime examples. The anatomical regions involved are often accessed via a retrosigmoid approach, which requires surgical expertise and the preservation of cranial nerve functions, aided by intraoperative neuromonitoring (IONM) techniques [6,7].

In this context, Izzo et al. (contribution 10) meticulously described the surgical protocol used for CPA surgery, focusing on the operating room setup, individualized surgical planning based on the patient’s anatomy and pathology using neuronavigation, and most importantly, IONM techniques and protocols. Detailing these techniques and explaining their function is essential to ensuring that the entire operating team speaks a mutually comprehensible language, optimizing collaboration during surgery, especially in cases of CPA tumors in which the anatomy may be distorted and the difficulty of recognizing structures is markedly increased.

Menna et al. (contribution 11) focused on pathologies strongly associated with neurovascular conflicts, specifically classical trigeminal neuralgia and hemifacial spasm. In their first research, the authors compared 76 elderly and non-elderly patients treated with microvascular decompression (MVD) in terms of clinical and surgical outcomes, dividing them into 2 groups based on age (over or under 65 years old). Notably, they found no significant differences between the groups in terms of acute pain relief (APR), the Barrow Neurological Index (BNI) at follow-up, complications, or the recurrence rate. Additionally, this study analyzed various factors as potential prognosticators of recurrence after MVD and found that the lack of a clear neurovascular conflict was the only independent predictor of TN recurrence after successful MVD surgery. These findings can be very useful in correctly placing the surgical indication in elderly patients, in whom the risk–benefit assessment of an MVD can often be complex.

The same group further investigated the lack of knowledge regarding factors associated with the recurrence of hemifacial spasm (HFS) after initially successful MVD surgery. HFS is considered primary when caused by neurovascular compression, typically due to the anterior inferior cerebellar artery (AICA). Given the limited efficacy of pharmacological treatments in these cases, MVD surgery is indicated, during which IONM techniques such as the assessment of the lateral spread response (LSR) are used [8,9]. LSR reveals that during spasms, the blink reflex spreads to muscles beyond the orbicularis oculi, likely due to antidromic impulse transmission between neighboring facial nerve fibers. During MVD surgery, continuous LSR monitoring is used to confirm adequate decompression. In their meta-analysis, Menna et al. (contribution 12) demonstrated that the persistence of LSR at the end of surgery was strongly associated with HFS recurrence after MVD. This finding provides a useful basis for further research on the need to enhance monitoring techniques, such as the ZL response [10], in this type of pathology.

Lastly, several articles in this Special Issue focused on important pathologies and approaches. Among these, Kasper et al. (contribution 13) analyzed data from a total of 1016 individual patients who underwent open microsurgical resection of olfactory groove meningiomas (OGMs) via pterional/unilateral, bifrontal with variations, and anterior interhemispheric approaches, examining the correlation between complications and the chosen surgical approach. A key finding was that unilateral approaches appear to have lower complication rates for OGM resection compared to bilateral approaches. However, the extent of resection was not consistently reported, making it difficult to identify differences. To address the lack of uniform data reporting and incorporate more recent translational studies, the authors developed a new classification system for OGMs aimed at improving comparability in reporting.

Luzzi et al. (contribution 14) provided a detailed report on the cranio-orbito-zygomatic (COZ) approach, an extension of the pterional approach involving orbitozygomatic (OZ) osteotomy, where each surgical step plays a precise role in target exposure and surgical freedom. Through a review of 55 articles, the authors outlined the main steps of the one-piece, two-piece (including the Zambraski and Al-Mefty variations), and three-piece techniques. As explained in the article, this approach requires specific tailoring depending on the location and size of the lesion, as well as the most suitable corridor for target exposure. It is particularly useful for basilar tip and anterior communicating artery (ACoA) aneurysms. While many authors advocate OZ osteotomy exclusively for lesions projecting upward, it has been shown that the increased surgical freedom provided by OZ osteotomy also significantly enhances the working space for lesions extending downward, thereby optimizing target exposure and reducing complication risks.

Another valuable perspective on the management of intra/extracranial-communicating jugular foramen lesions, particularly intra/extracranial-communicating jugular foramen paragangliomas (IECJFPs), was provided by Li et al. (contribution 15), who presented a case report treated with a two-stage surgery. First, the intracranial portion of the paraganglioma was addressed via a retrosigmoid approach. Following the expansion of the extracranial portion, a second surgery was performed using the infratemporal approach. While initial surgery for the extracranial tumor followed by a second procedure for the intracranial tumor is appropriate in IECJFP patients with low cranial nerve deficits and middle ear involvement, for cases without lower cranial nerve deficits or tympanic cavity involvement, it is preferable to perform the first surgery for the intracranial lesion along with subtotal resection of the extracranial tumor in the second surgery, as outlined by the authors.

Finally, Matoušek et al. (contribution 16) provided a critical example of the management of iatrogenic injury to the internal carotid artery (ICA), a rare and likely underreported complication of transnasal endoscopic skull base surgery. Although treatment algorithms have been suggested, there is no definitive consensus or guideline for managing this severe complication. The authors, reporting a case of ICA injury during transsphenoidal biopsy of a tumor in the cavernous sinus, described a highly effective treatment algorithm for managing this complication, proposing an effective method of managing these potentially fatal injuries and highlighting the need to optimize their surgical and postoperative management.

## 3. Conclusions

The scientific articles published in this Special Issue are crucial to gaining a better understanding of future developments in the field of skull base surgery, as they provide valuable insights into the latest advancements, techniques, and complications associated with this highly complex and challenging specialty. These publications not only emphasize the importance of innovative approaches, but also highlight the critical role of multidisciplinary collaboration in improving surgical outcomes. Through systematic analyses, reviews, and case studies, the articles contribute to the refinement of surgical techniques, offer guidance on managing complications like CSF leaks and nerve injuries, and foster the development of new technologies such as machine learning in neurosurgery. These findings collectively enhance the understanding of skull base surgery, paving the way for improved patient care before, during, and after surgery.
